# Biphasic Calcium Phosphate (BCP)-Immobilized Porous Poly (d,l-Lactic-*co*-Glycolic Acid) Microspheres Enhance Osteogenic Activities of Osteoblasts

**DOI:** 10.3390/polym9070297

**Published:** 2017-07-21

**Authors:** Kyu-Sik Shim, Sung Eun Kim, Young-Pil Yun, Somang Choi, Hak-Jun Kim, Kyeongsoon Park, Hae-Ryong Song

**Affiliations:** 1 Department of Biomedical Science, College of Medicine, Korea University, Anam-dong, Seongbuk-gu, Seoul 02841, Korea; breakdown88@nate.com (K.-S.S.); chlthakd1029@naver.com (S.C.); 2 Department of Orthopedic Surgery and Rare Diseases Institute, Korea University Medical College, Guro Hospital, #80, Guro-dong, Guro-gu, Seoul 08308, Korea; sekim10@korea.ac.kr (S.E.K.); ofeel0479@korea.ac.kr (Y.-P.Y.); dakjul@korea.ac.kr (H.-J.K.); 3 Department of Systems Biotechnology, College of Biotechnology and Natural Resources, Chung-Ang University, 4726 Seodong-daero, Daedeok-myeon, Anseong-si, Gyeonggi-do 17546, Korea

**Keywords:** porous microspheres (PMSs), biphasic calcium phosphate (BCP), MG-63 cells, osteogenic activity

## Abstract

The purpose of this study was to evaluate the potential of porous poly (d,l-lactic-*co*-glycolic acid) (PLGA) microspheres (PMSs) immobilized on biphasic calcium phosphate nanoparticles (BCP NPs) (BCP-IM-PMSs) to enhance osteogenic activity. PMSs were fabricated using a fluidic device, and their surfaces were modified with l-lysine (aminated-PMSs), whereas the BCP NPs were modified with heparin–dopamine (Hep-DOPA) to obtain heparinized–BCP (Hep-BCP) NPs. BCP-IM-PMSs were fabricated via electrostatic interactions between the Hep-BCP NPs and aminated-PMSs. The fabricated BCP-IM-PMSs showed an interconnected pore structure. In vitro studies showed that MG-63 cells cultured on BCP-IM-PMSs had increased alkaline phosphatase activity, calcium content, and mRNA expression of osteocalcin (OCN) and osteopontin (OPN) compared with cells cultured on PMSs. These data suggest that BCP NP-immobilized PMSs have the potential to enhance osteogenic activity.

## 1. Introduction

To regenerate bone defects, bone grafts such as autografts and allografts are widely used. These approaches lead to strong autograft osteoinduction, osteoconduction, and osteogenesis as well as allograft osteoinduction and osteoconduction [1]. However, bone grafts have some disadvantages in terms of pain, donor site morbidity, rejection, and disease transmission [2,3]. Given these problems, bone tissue engineering involving a three-dimensional (3D) porous scaffold, stem cells, and cellular stimulation (e.g., via growth factors or drugs) has become a recent focus of interest. Such engineering could potentially help with regeneration or repair of bone defects [4]. 3D porous scaffolds provide the structural support for cell adhesion, proliferation, and differentiation. They also act as substrates for induction of new bone formation at the defect or injury site through surgical procedures [5,6]. However, it is difficult to regenerate or repair bone defects in non-invasive or minimally invasive surgical procedures using 3D porous scaffolds.

From a clinical perspective, injectable scaffolds are a desirable alternative substrate for tissue regeneration of irregular-shaped bone defects since only minimally invasive surgical procedures are required. Moreover, injectable scaffolds have the advantages of small scars, short operation times, and lower risk of infection since only 10–16 gauge needles are used [7,8]. Many injectable scaffolds have been investigated, among which microspheres are generally believed to have the most utility as vehicles for sustained drug or protein delivery due to their inherently small size, small volume, large specific surface area, and high drug loading efficiency [9,10,11]. Porous microspheres have been determined to be suitable carriers for cells, drugs, and proteins due to their interconnected structures extending between external surface pores and internal core pores [12,13]. However, naked microspheres or porous microspheres (i.e., without cells, proteins, drugs, or osteoconductive material) have potentially limited ability to replace or regenerate bone tissue.

Bioceramics such as hydroxyapatite (HAp), calcium carbonate, calcium sulfate, and β-tricalcium phosphate (β-TCP) have been used for the treatment of bone defects for over 30 years [14]. Biphasic calcium phosphate (BCP) ceramics are related to a group of bone substitute materials consisting of mixtures of HAp and β-TCP in fixed ratios [15,16]. These materials have been shown to possess excellent osteoconductivity, bioactivity, biocompatibility, and biodegradability [17,18,19]. Although BCP ceramics have many advantages, their intrinsic brittleness makes them unsuitable in calcium phosphate-based scaffolds with complex structures [20,21,22]. To overcome these drawbacks, composite materials have been pursued. Compared with ceramic and polymers, these composite materials have been shown to have better mechanical properties and osteogenic activity [22,23]. Polymer/ceramic composite scaffolds have been fabricated by chemical foaming, solvent casting, particle/salt leaching, and thermally-induced phase separation [24,25,26,27,28]. However, polymer/ceramic composite scaffolds also have several limitations, including the presence of large aggregates and limited exposure of ceramics on the scaffold surface. As an alternative approach, ceramics have been coated on the surfaces of polymer scaffolds using simulated body fluid (SBF) [23,29,30]. The SBF coating method requires a time-consuming incubation and maintenance of SBF pH, which limits the effectiveness of this approach to generate bioactive bone-like apatite layers on the scaffold surface. A previous study conducted by Kim et al. demonstrated that nano-hydroxyapatite (N-HAp) grafted with anionic poly(ethylene glycol methacrylate phosphate, PolyEGMP) was uniformly immobilized on a chitosan scaffold surface [31]. In addition, these scaffolds were shown to have strong osteogenic potential based on evaluation of alkaline phosphate (ALP) activity in vitro and animal studies. More recently, HAp nanoparticles (NPs) modified with heparin-dopamine (Hep-DOPA) and lactoferrin (LF) were shown to significantly increase alkaline phosphatase (ALP) activity, calcium content, and mRNA expression of osteocalcin (OCN) and osteopontin (OPN) compared with HAp and Hep–HAp NPs [32].

Based on these previous studies, we fabricated porous microspheres (PMSs) using a fluidic device and then introduced positively charged amine groups onto the surfaces of the PMSs through modification with l-lysine. Additionally, Hep-DOPA was anchored on the surface of BCP NPs to introduce negative charge. Finally, Hep-BCP NPs modified with Hep-DOPA were immobilized on the surface of the aminated-PMSs modified with l-lysine in the MES buffer solution, thereby fabricating BCP-immobilized PMSs (BCP-IM-PMSs). The objective of this study was to investigate the potential of BCP-IM-PMSs to enhance osteogenic activity. Hence, we investigated alkaline phosphatase (ALP) activity, calcium content, and gene expression in vitro. Also, we compared it to in vitro osteogenic activities of unmodified PMSs and BCP-mixed PMSs (BCP-IM-PMSs). 

## 2. Materials and Methods

### 2.1. Materials

Poly (d,l-lactic-*co*-glycolic acid) (PLGA, 50:50, molecular weight: 30,000–60,000), poly vinyl alcohol (PVA, molecular weight: 13,000–23,000, 98% hydrolyzed), dichloromethane (DCM), gelatin from porcine skin, ascorbic acid, dexamethasone, and β-glycerophosphate were purchased from Sigma-Aldrich (St. Louis, MO, USA). Dulbecco’s modified Eagle’s medium (DMEM), fetal bovine serum (FBS), phosphate-buffered saline (PBS), and penicillin-streptomycin were obtained from Gibco BRL (Rockville, MD, USA). Cell counting kit-8 (CCK-8) reagents were obtained from Dojindo, Inc. (Kumamoto, Japan). Biphasic calcium phosphate (BCP; Hydroxyapatite = 60%, Tricalcium phosphate = 40%) nanoparticles were kindly donated by Ossgen Corporation (Gyeongbuk, Korea). MG-63 cells (human osteosarcoma cell line) were obtained from the Korea Cell Line Bank (KCLB No. 21427, Seoul, Korea). All chemical reagents were of the purest analytical grade available.

### 2.2. Fabrication of Porous PLGA Microspheres (PMSs), BCP-Mixed PMSs, and BCP-Immobilized PMSs

**Figure 1:**
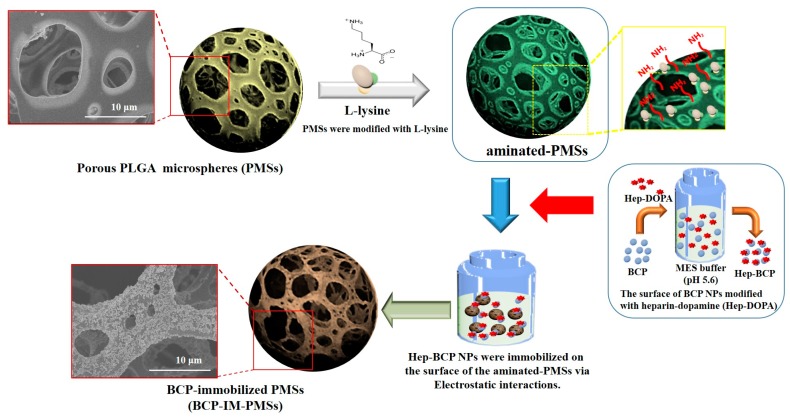
Schematic diagram of the fabrication of biphasic calcium phosphate (BCP)-immobilized porous microspheres (BCP-IM-PMSs). PMSs were fabricated using a fluidic device. Next, the fabricated PMSs were treated with l-lysine, yielding aminated-PMSs. BCP nanoparticles (NPs) were modified with heparin-dopamine (Hep-DOPA) to obtain Hep-BCP NPs. Finally, BCP-immobilized PMSs (BCP-IM-PMSs) were fabricated by immobilization of Hep-BCP NPs on the surface of the aminated-PMSs via electrostatic interactions.

To fabricate the porous PLGA microspheres (PMSs), BCP-mixed PMSs (BCP-M-PMSs), and BCP-immobilized PMSs (BCP-IM-PMSs), a fluidic device was used as described in our previous work [12,13]. Briefly, to fabricate the BCP-mixed PMSs, BCP NPs (37.8 mg) at a given weight ratio of PLGA to BCP (27 wt %) were dispersed in DCM. PLGA (140 mg) was added to DCM with BCP NPs. Gelatin (7.5 wt %) and PVA (2 wt %) were also added to the BCP/PLGA solution. A homogenizer (Ultra-Turrax T-25 Basic, IKA, Staufen im Breisgau, Germany) was used for 1 min to create a water-in-oil (W/O) polymer emulsion. The W/O emulsion is hereafter referred to as the discontinuous phase, while the PVA solution is hereafter referred to as the continuous phase. The discontinuous and continuous phases had flow rates of 0.06 and 0.65 mL/min, respectively. The BCP-M-PMSs were immersed in warm water at 40 °C under gentle stirring for 1 h to remove residual gelatin, after which the BCP-M-PMSs were washed with PBS and lyophilized for 3 days. To fabricate the BCP-IM-PMSs, the surfaces of the BCP NPs were first modified with Hep-DOPA to anchor the negatively charged groups. In brief, BCP NPs were immersed in 10 mM Tris·HCl (pH 8.0) and then dissolved in Hep-DOPA. This reaction was allowed to proceed for 24 h in the dark. After the reaction, Hep-anchored BCP (Hep-BCP) NPs were rinsed with distilled water (DW) and lyophilized for 3 days. The particle size and zeta potential value of Hep-BCP NPs were 333.17 ± 17.79 nm and −12.72 ± 1.24 mV, respectively. Hep-BCP NPs were well dispersed in aqueous solution due to surface immobilization of negatively charged heparin on BCP NPs. The surface of the PMSs was also modified by l-lysine to anchor the positively-charged groups. l-lysine (2 mg/mL) was dissolved in 0.1 M MES buffer (pH 5.6). Then, PMSs were added to the aforementioned l-lysine solutions and shaken gently overnight. The l-lysine-anchored PMSs are hereafter referred to as aminated-PMSs. To immobilize the Hep-BCP NPs on the surfaces and inside the PMSs, Hep-BCP NPs (37.8 mg) [weight ratio PLGA:BCP (27 wt %)] were dispersed in 0.1 M MES buffer (pH 5.6) using a bath-type ultra sonicator (Powersonic 405, Hwashin Inc., Soul, Korea) for 3 h at 350 W and 4 °C. The aminated-PMSs were then immersed in the Hep-BCP solution. The BCP-immobilized PMSs were washed with DW and lyophilized for 3 days. The BCP-immobilized PMSs are hereafter referred to as BCP-IM-PMSs (Figure 1).

### 2.3. Characterization of the PMSs, Aminated-PMSs, BCP-M-PMSs, and BCP-IM-PMSs

The morphologies and elemental compositions of the PMSs, aminated-PMSs, BCP-M-PMSs, and BCP-IM-PMSs were determined using a field emission scanning electron microscope (FE-SEM, S-2300, Hitachi, Tokyo, Japan) with energy dispersive X-ray spectroscopy (EDX). To this end, the specimens were coated with gold using a sputter-coater (Eiko IB, Tokyo, Japan), and the FE-SEM was operated at 3 kV. To evaluate the average pore size of each group, selected (*n* = 50) porous PMSs, aminated-PMSs, BCP-M-PMSs, and BCP-IM-PMSs were randomly determined using Image J analysis software (Softonic International, R.B., Barcelona, Spain) based on FE-SEM images. The surface chemical compositions of the PMSs, aminated-PMSs, BCP-M-PMSs, and BCP-IM-PMSs were analyzed by an X-ray photoelectron spectroscopy (XPS) K-Alpha spectrometer (Thermo Electron, Rockford, IL, USA) at the Korea Basic Science Institute Busan Center (Busan, Korea).

### 2.4. Cell Proliferation

CCK-8 reagents were used to assess cell proliferation as previously described [12,13,32]. Briefly, MG-63 cells were carefully seeded at a concentration of 1 × 10^5^ cells/mL on 10 mg of PMSs, aminated-PMSs, BCP-M-PMSs, and BCP-IM-PMSs in 24-well tissue culture plates. Cells were maintained in DMEM supplemented with 10% FBS and 1% antibiotics (100 U/mM penicillin, 0.1 mg/mL streptomycin). CCK-8 reagents were added to each sample and incubated for 1 h after 3 and 7 days of incubation. Cell proliferation was determined by measuring the absorbance of each well at 450 nm using a Flash Multimode Reader (Varioskan™, Thermo, 3001–2019, Grand Island, NY, USA).

### 2.5. Alkaline Phosphatase (ALP) Activity

MG-63 cells were obtained from Korea Cell Line Bank (No. 21427, Seoul, Korea). In this study, we used MG-63 cells because they are commonly used to evaluate osteogenic evaluation of bioceramices, bioactive drugs, peptides, and proteins in/on various scaffolds. 

MG-63 cells were carefully seeded at a density of 1 × 10^5^ cells/mL on 10 mg of PMSs, aminated-PMSs, BCP-M-PMSs, or BCP-IM-PMSs in 24-well tissue culture plates. Cells were cultured in DMEM supplemented with 10% FBS, 50 μg/mL ascorbic acid, 10 nM dexamethasone, and 10 mM β-glycerophosphate with 100 U/mL penicillin and 100 μg/mL streptomycin for 10 days. At predetermined time intervals (3, 7, and 10 days), each sample was rinsed with PBS, and 1× RIPA buffer was added for cell lysis. To remove cell debris, cell lysates were centrifuged at 13,500 rpm for 1 min. After centrifugation, *p*-nitrophenyl phosphate solution was added to the supernatants. The reactions were incubated for 30 min at 37 °C and then stopped with 1 M NaOH. Based on the standard of *p*-nitrophenyl phosphate solution, the ALP activity was determined by converting *p*-nitrophenyl phosphate to *p*-nitrophenol. ALP activity was evaluated using a Flash Multimode Reader to measure the absorbance at 405 nm.

### 2.6. Calcium Deposition

MG-63 cells (1 × 10^5^ cells/well) were carefully seeded on 10 mg of PMSs, aminated-PMSs, BCP-M-PMSs, or BCP-IM-PMSs in 24-well tissue culture plates. At predetermined time intervals (days 7 and 21), 0.5 M HCl (400 μL) was added to the cell/substrate mixtures. After centrifugation, calcium deposition in the supernatants was measured using a QuantiChrom Calcium Assay Kit (DICA-500, BioAssay Systems, Hayward, CA, USA) according to the manufacturer’s instructions. Calcium chloride was used as a standard. The amount of deposited calcium was determined using a Flash Multimode Reader to measure the absorbance at 612 nm.

### 2.7. Gene Expression

To quantify the mRNA expression levels of the osteogenic differentiation markers osteocalcin (OCN) and osteopontin (OPN), quantitative real-time polymerase chain reaction (RT-PCR) was performed on days 7 and 21. MG-63 cells (1 × 10^5^ cells/well) were seeded on 10 mg of PMSs, aminated-PMSs, BCP-M-PMSs, or BCP-IM-PMSs and cultured in 24-well tissue culture plates. Total RNA (1 μg) was used for cDNA synthesis with AccuPower RT PreMix (Bioneer, Daejeon, Korea). The following primers were used: osteocalcin (OCN): (F) 5′-TTG GTG CAC ACC TAG CAG AC-3′, (R) 5′-ACC TTA TTG CCC TCC TGC TT-3′; osteopontin (OPN): (F) 5′-GAG GGC TTG GTT GTC AGC-3′, (R) 5′-CAA TTC TCA TGG TAG TGA GTT TTC C-3′; and glyceraldehyde 3-phosphate dehydrogenase (GAPDH) (F) 5′-ACT TTG TCA AGC TCA TTT CC-3′, (R) 5′-TGC AGC GAA CTT TAT TGA TG-3′. PCR amplification and detection were carried out on an ABI7300 Real-Time Thermal Cycler (Applied Biosystems, Foster, CA, USA) using a DyNAmoTM SYBR^®^ Green qPCR Kit (Finnzymes, Espoo, Finland). OCN and OPN mRNA expression levels were normalized to those of GAPDH and are expressed as relative values. 

### 2.8. Statistical Analysis

Quantitative data are presented as mean ± standard deviation. Data were compared between groups via one-way ANOVA using SYSTAT software (Chicago, IL, USA). Differences were considered to be statistically significant at * *p* < 0.05 and ** *p* < 0.01.

## 3. Results

### 3.1. Characterization of PMSs, aminated–PMSs, BCP-M-PMSs, and BCP-IM-PMSs

**Figure 2:**
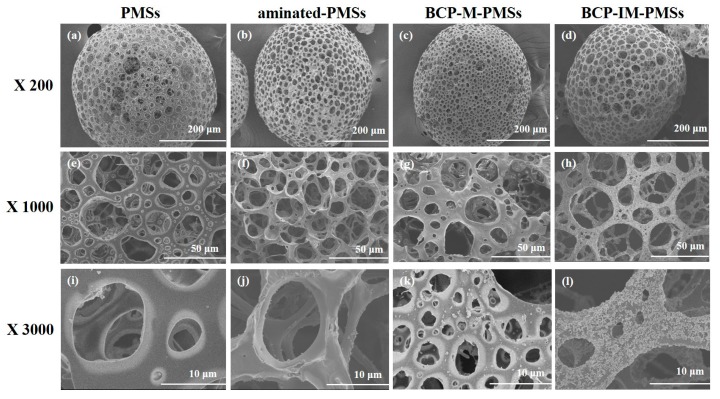
Scanning electron microscope (SEM) images of porous microspheres (PMSs) (**a**,**e**,**i**), aminated-PMSs (**b**,**f**,**j**), BCP-mixed PMSs (BCP-M-PMSs) (**c**,**g**,**k**), and BCP-IM-PMSs (**d**,**h**,**l**).

Figure 2 shows the morphologies and sizes of the PMSs, aminated-PMSs, BCP-M-PMSs, and BCP-IM-PMSs as determined by FE-SEM. The fabricated PMSs, aminated-PMSs, BCP-M-PMSs, and BCP-IM-PMSs were round and exhibited interconnected porous structures in their interiors and on their surfaces. The BCP-M-PMSs and BCP-IM-PMSs both displayed coarse surfaces, whereas the PMSs and aminated-PMSs had smooth surfaces. Additionally, the surfaces of the BCP-IM-PMSs were rougher than those of the BCP-M-PMSs. The average pore sizes were 24 ± 7 μm for PMSs, 19 ± 6 μm for aminated-PMSs, 22 ± 11 μm for BCP-M-PMSs, and 23 ± 7 μm for BCP-IM-PMSs. EDX analysis was also used to confirm the elemental compositions of the PMSs, aminated-PMSs, BCP-M-PMSs, and BCP-IM-PMSs. Successful anchoring of l-lysine on the surface of and inside the PMSs was confirmed by the increased N content of the aminated-PMSs compared with that of the naked PMSs. The BCP-M-PMSs and BCP-IM-PMSs were characterized by increased P and Ca contents. In particular, the BCP-IM-PMSs had lower N content compared to aminated PMSs, and showed higher P and Ca contents than the BCP-M-PMSs (Table 1), indicating that the negatively charged Hep-BCP NPs were successfully immobilized on the aminated PMSs. Next, the surface chemical compositions of the PMSs, aminated-PMSs, BCP-M-PMSs, and BCP-IM-PMSs were evaluated by XPS (Table 2). In the l-lysine-anchored PMSs, the N1 content was increased compared to that of the PMSs. In addition, the P2p and Ca2p contents of both the BCP-M-PMSs and BCP-IM-PMSs were greater than those of the aminated-PMSs. Furthermore, the P2p and Ca2p contents of the BCP-IM-PMSs were 0.87% to 1.02% and 1.91% to 3.87% greater, respectively, than those of the BCP-M-PMSs.

### 3.2. Cell Proliferaiton

**Figure 3:**
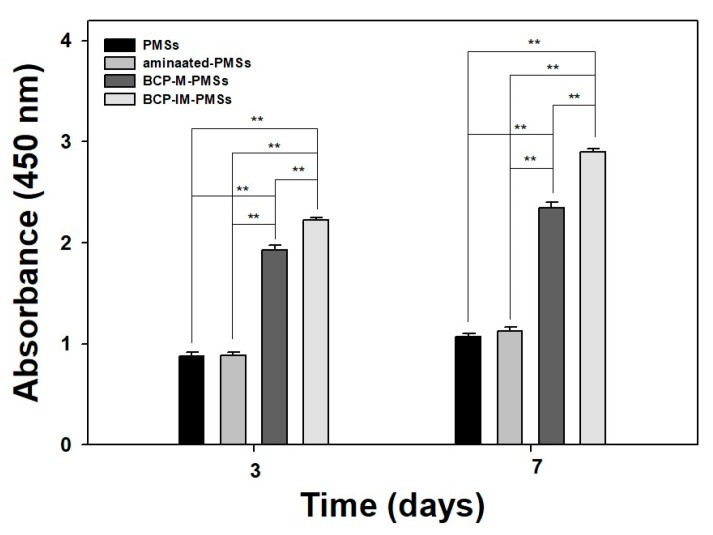
Proliferation of MG-63 cells cultured on PMSs, aminated-PMSs, BCP-M-PMSs, or BCP-IM-PMSs for 3 or 7 days (*n* = 5) (* *p* < 0.05 and ** *p* < 0.01).

After 3 and 7 days of incubation, the proliferation of MG-63 cells grown on PMSs, aminated-PMSs, BCP-M-PMSs, or BCP-IM-PMSs was investigated (Figure 3). In our previous study, we already confirmed with confocal laser scanning microscope that the carefully seeded cells were attached in/on the PMSs [33]. MG-63 cells grown on PMSs with BCP exhibited significantly greater proliferation than cells grown on PMSs or on aminated-PMSs at days 3 and 7 (** *p* < 0.01). Moreover, MG-63 cell proliferation on BCP-IM-PMSs was significantly different than that of cells on BCP-M-PMSs on days 3 and 7 (** *p* < 0.01).

### 3.3. ALP Activity

**Figure 4:**
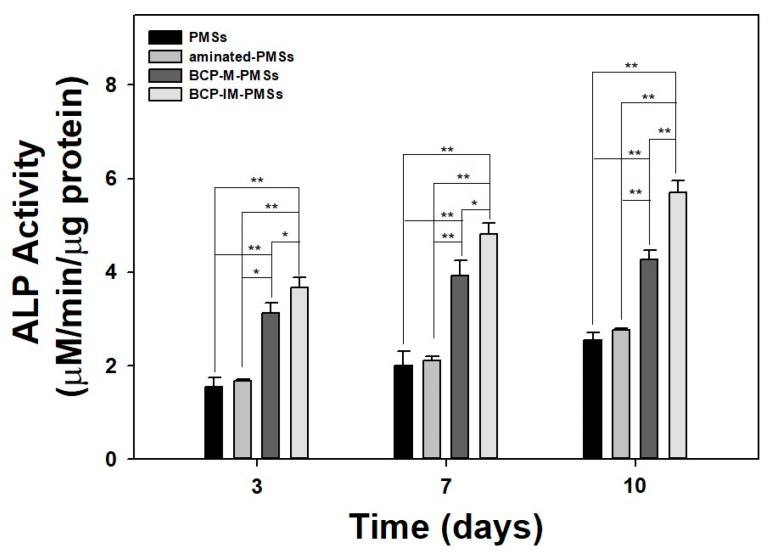
Alkaline phosphatase (ALP) activity of MG-63 cells cultivated on PMSs, aminated-PMSs, BCP-M-PMSs, or BCP-IM-PMSs after 3, 7, or 10 days of incubation (*n* = 5) (* *p* < 0.05 and ** *p* < 0.01).

The ALP activity of MG-63 cells cultured on PMSs, aminated-PMSs, BCP-M-PMSs, or BCP-IM-PMSs was determined after 3, 7, and 10 days (Figure 4). At day 3, the ALP activity of MG-63 cells cultured on BCP-M-PMSs or BCP-IM-PMSs was significantly different from that of cells cultured on PMSs alone (** *p* < 0.01). At day 7, the ALP activity of MG-63 cells grown on BCP-M-PMSs or BCP-IM-PMSs was significantly increased compared to that of MG-63 cells cultured on PMSs (** *p* < 0.01). At 10 days, MG-63 cells grown on BCP-M-PMSs or BCP-IM-PMSs had significantly greater ALP activity than cells grown on PMSs (** *p* < 0.01). Additionally, MG-63 cells grown on BCP-IM-PMSs had significantly higher ALP activity than MG-63 cells grown on BCP-M-PMSs at days 3, 7, and 10 days (* *p* < 0.05 and ** *p* < 0.01, respectively). However, ALP activity was not markedly different on days 3, 7, and 10 for MG-63 cells grown on PMSs versus cells grown on aminated-PMSs.

### 3.4. Calcium Deposition

**Figure 5:**
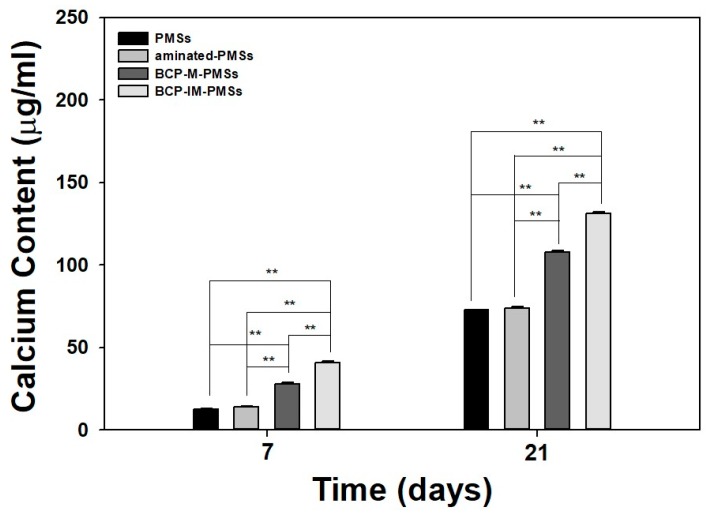
Amount of calcium deposited by MG-63 cells grown on PMSs, aminated-PMSs, BCP-M-PMSs, or BCP-IM-PMSs after 7, 14, or 21 days of incubation (*n* = 5) (* *p* < 0.05 and ** *p* < 0.01).

Figure 5 shows the amounts of calcium deposited by MG-63 cells cultivated on PMSs, aminated-PMSs, BCP-M-PMSs, or BCP-IM-PMSs after 7 or 21 days of incubation. On day 7, MG-63 cells cultured on BCP-IM-PMSs had deposited significantly greater calcium than MG-63 cells grown on PMSs (** *p* < 0.01). On day 21, MG-63 cells cultured on BCP-IM-PMSs had deposited markedly more calcium compared with cells cultured on PMSs (** *p* < 0.01). Moreover, MG-63 cells cultured on BCP-IM-PMSs had deposited significantly different amounts of calcium on days 7 and 21 compared to cells cultured on BCP-M-PMSs (** *p* < 0.01).

### 3.5. Gene Expression

**Figure 6:**
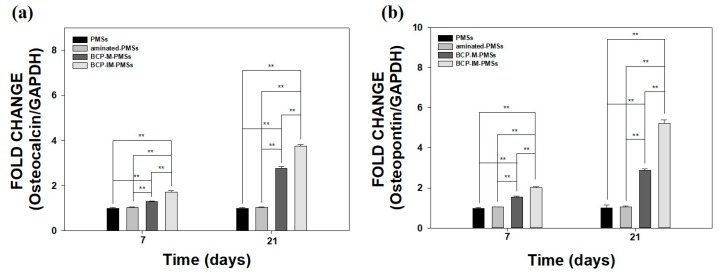
Real-time PCR analysis of (**a**) osteocalcin (OCN) and (**b**) osteopontin (OPN) expression in MG-63 cells after 7 or 21 days of incubation with PMSs, aminated-PMSs, BCP-M-PMSs, or BCP-IM-PMSs (*n* = 5) (* *p* < 0.05 and ** *p* < 0.01).

After 7 and 21 days of incubation, real-time PCR was used to determine the mRNA expression levels of OCN and OPN in MG-63 cells cultured on PMSs, aminated-PMSs, BCP-M-PMSs, or BCP-IM-PMSs (Figure 6). As shown in Figure 6a,b, OCN and OPN mRNA expression levels were significantly different at days 7 and 21 between MG-63 cells grown on BCP-M-PMSs or BCP-IM-PMSs compared with those grown on PMSs (** *p* < 0.01). In addition, OCN and OPN expression levels were markedly higher on days 7 and 21 in MG-63 cells grown on BCP-IM-PMSs than in cells grown on BCP-M-PMSs (** *p* < 0.01). However, the OCN and OPN expression levels in MG-63 cells cultured on PMSs and MG-63 cells cultured on aminated-PMSs were not significantly different at any time-point.

## 4. Discussion

The purpose of this study was to investigate the potential of BCP-IM-PMSs to enhance osteogenic activity through in vitro studies. PMSs, which have porous structures, are believed to be good environments for fostering cell attachment, proliferation, and differentiation [34,35]. In the conventional method, the porogen ammonium bicarbonate (NH_4_HCO_3_) is added to the appropriate solution for PMS preparation in a water-in-oil-in-water (W/O/W) emulsion system [35,36]. The porogen is removed by solvent casting/particulate leaching, thereby forming interconnective pores in both the surface and interior. However, porogen-derived PMSs have inherent restrictions. For example, the high-shear natural method of emulsification generates microspheres with heterogeneous sizes and morphologies [34,35]. In addition, it is time-consuming to completely eliminate the porogens [34,37]. In this study, we fabricated PMSs with or without BCP with interconnected pores using a fluidic device. The SEM images demonstrated that the fabricated PMSs with or without BCP had spherical structures and open surface and interior pores. Our previous studies demonstrated that PMSs made from fluidic devices are round, with interconnected surfaces and interior pores [12,13]. In addition, high magnification SEM images revealed that both PMSs and aminated-PMSs had relatively smooth surfaces. On the other hand, PMSs with BCP NPs had rough surfaces. Moreover, BCP-IM-PMSs had much rougher surfaces than BCP-M-PMSs because the BCP NPs were immobilized on the surface of the PMSs. Chitosan scaffolds with immobilized HAp NPs were previously shown to have rougher, more uniform surfaces compared with HAp NP-mixed chitosan scaffolds [31]. Notably, the SEM results of Kim et al. [31] are consistent with our results. 

To fabricate BCP-IM-PMSs, Hep-DOPA was used to easily modify the BCP NP surface. This step utilizes the marine mussel’s adhesive mechanism [38]. DOPA, which harbors ortho-dihydroxyphenyl (catechol) functional groups, readily changes different materials including organic, inorganic, and metallic materials in alkaline solutions (pH 8.0) [38]. Our previous studies reported that Hep-DOPA molecules were readily immobilized on the surface of PMS, hydroxyapatite (HAp), and titanium substrates in basic environments [12,13,32,39]. Thus, based on our previous studies, we chose to immobilize Hep-DOPA molecules on the surfaces of the BCP NPs. In the dispersion test, the BCP NPs modified with Hep-DOPA were well-dispersed in aqueous conditions, whereas pure BCP NPs were rapidly precipitated in aqueous conditions within 30 min (data not shown). Consistent with our previous report, HAp NPs with adherent Hep-DOPA molecules were well-diffused in aqueous conditions for up to 6 h and maintained turbidity for 24 h. However, pure HAp NPs were quickly precipitated in 1 h due to extensive aggregation [32]. These results suggest that heparin-immobilized BCP or HAp NPs prohibit the aggregation of BCP or HAp NPs, possibly due to the repulsion of heparin molecules, which have a high content of negatively-charged sulfate and carboxyl groups. Thus, we chose to fabricate BCP NP-immobilized PMSs because we hypothesized that they might remain well-dispersed during surface immobilization on aminated-PMSs. 

The EDX and XPS results showed that PMSs with anchored l-lysine showed increased nitrogen (N) content compared with naked PMSs. Lee and colleagues [40] similarly confirmed that an l-lysine–grafted titanium (Ti) substrate had increased nitrogen content compared with the Ti substrate only. Phosphorus and calcium were found in both BCP-M-PMSs and BCP-IM-PMSs; however, BCP-IM-PMSs showed higher phosphorus and calcium levels than BCP-M-PMSs. This result suggests that the Hep-BCP NPs immobilized on the surface of the aminated-PMSs via electrostatic interactions are more surface-exposed than the PMSs with the BCP NPs via the mixing method. In one previous study, chitosan scaffolds with surface-immobilized HAp were reported to show increased phosphorus and calcium contents compared with HAp-mixed chitosan scaffolds [31]. The XPS result of this study is consistent with our results.

To evaluate the osteogenic capabilities of the PMSs, aminated-PMSs, BCP-M-PMSs, and BCP-IM-PMSs, ALP activity, calcium content, and osteocalcin (OCN) and osteopontin (OPN) expression were measured in MG-63 cells. At days 3 and 7, MG-63 cells grown on BCP-M-PMSs or BCP-IM-PMSs exhibited significantly increased proliferation compared to those grown on PMSs (** *p* < 0.01). Moreover, MG-63 cells cultivated on BCP-IM-PMSs exhibited markedly greater proliferation on days 3 and 7 than those cultivated on BCP-M-PMSs (** *p* < 0.01). However, MG-63 cells grown on PMSs and aminated-PMSs exhibited similar proliferation during the 7-day culture period. Cells cultured on scaffolds with surface-immobilized nano-HAp exhibited significantly greater proliferation than cells cultured on scaffolds alone or N-HAp-mixed scaffolds [23,31]. These results imply that surface-placed nanoscale ceramics are key for optimal cell adhesion and proliferation.

As key components of bone matrix vesicles and mineralization, ALP activity and calcium content are often evaluated as markers of early and late osteogenic differentiation, respectively [12,13,39,40]. The ALP activity of MG-63 cells cultured on PMSs, aminated-PMSs, BCP-M-PMSs, or BCP-IM-PMSs was increased for culture times up to 10 days. Furthermore, ALP activity was significantly higher on days 3, 7, and 10 in cells cultured on PMSs with BCP NPs compared with cells cultured on PMSs alone. Moreover, significant differences in ALP activity were observed between MG-63 cells cultured on BCP-IM-PMSs versus those cultured on BCP-M-PMSs. Consistent with the ALP activity results, the calcium contents of MG-63 cells grown on BCP-M-PMSs or BCP-IM-PMSs were significantly greater than those of cells grown on PMSs. Additionally, MG-63 cells cultured on BCP-IM-PMSs had significantly different calcium content compared with cells cultured on BCP-M-PMSs. Kim et al. [29] demonstrated that HAp-coated PLGA scaffolds modified by incubation with 5× simulated body fluid (SBF) solution showed increased ALP activity and calcium deposition of osteoblast cells compared with unmodified PLGA scaffolds. Moreover, Kim et al. [31] confirmed that human adipose-derived stem cells (hADSCs) cultured on HAp-immobilized chitosan scaffolds showed significantly different ALP activity compared to those cultured on chitosan scaffolds alone. Taken together, these results demonstrate that BCP/HAp-immobilized and BCP/HAp-coated scaffolds directly affect both early and late differentiation of osteoblast cells and stem cells.

To evaluate osteogenic differentiation, we performed real-time PCR to measure the gene expression of OCN and OPN after 7 and 21 days of incubation. Expression of OCN and OPN is known to be upregulated in osteoblast-like cells after stimulation of osteogenic differentiation [12,39,40]. At days 7 and 21, MG-63 cells cultured on PMSs with BCP NPs exhibited significantly higher OCN and OPN expression than cells cultured on PMSs. Moreover, the OCN and OPN mRNA levels in MG-63 cells grown on BCP-IM-PMSs were markedly higher than those in cells grown on BCP-M-PMSs. These results are consistent with the ALP activity and calcium content findings. The mRNA levels of OCN, OPN, and Runt-related transcription factor2 (*Runx2*) in rat bone marrow stem cells (BMSCs) grown on hydroxyapatite-coated genipin-chitosan conjugation scaffolds (HGCCS) have been shown to be significantly higher than those in cells grown on a genipin cross-linked chitosan framework (GCF) [41]. These results indicate that BCP immobilization and HAp coating on the PMS or scaffold surface both directly promote osteogenic differentiation of osteoblast cells and stem cells.

Recently, interest has increased in using polymer/ceramic composite microspheres as injectable scaffolds in bone regeneration. These composite microspheres have many advantages, including the osteoconductive properties of ceramics and a favorable environment for cell adhesion, proliferation, and differentiation. In this study, we showed that PMSs and aminated-PMSs had negligible osteogenic effects and mineralization, and the surface modification of PMSs with BCP NPs could more greatly promote the osteogenic activities of MG-63 cells than mixing of BCP NPs within PMSs. These four types of microspheres have similar pore sizes. However, BCP-IM-PMSs showed the highest osteogenic activities compared to other PMSs because surface immobilized BCP NPs on PMSs was similar to the minerals of natural bone to favor cell differentiation, and the released calcium and phosphate ions after degradation of BCP NPs might facilitate the formation of the biological apatite on scaffolds [42]. This might indicate that surface immobilization of BCP NPs on PMSs leads to the enhanced osteogenesis effects rather than pore sizes of PMSs. Thus, we confirmed that that BCP-IM-PMSs markedly enhanced osteogenic activity compared to BCP-M-PMSs. The surface of porous microspheres can be easily modified with BCP NPs and also used as delivery carriers of many kinds of drugs (i.e., anti-cancer drugs, antibiotics, anti-inflammatory drugs, and bone stimulating medications). Thus, porous microspheres with multifunctional properties can be broadly used to treat fractures, tumors, and inflammatory diseases. For their clinical application, future studies must be performed in animal models with bone defects associated with fractures, tumors, and inflammation.

## 5. Conclusions

We fabricated PMSs using a fluidic device. The fabricated PMSs were modified with l-lysine and showed a positively-charged surface. The BCP NPs were also modified by Hep-DOPA to obtain Hep-BCP NPs, which had a negatively-charged surface. Next, Hep-BCP NPs were immobilized on the surface of the aminated-PMSs via electrostatic interactions to obtain BCP-IM-PMSs. We demonstrated that MG-63 cells cultured with BCP-IM-PMSs showed significantly higher ALP activity, calcium deposition, and expression of osteogenic differentiation genes (OCN and OPN) compared with cells cultured with PMSs or BCP-M-PMSs. Therefore, we suggest that BCP-immobilized porous microspheres can be used to enhance osteogenic activity.

## Figures and Tables

**Table 1:** Elemental compositions of PMSs, aminated PMSs, BCP-M-PMSs, and BCP-IM-PMSs.

Samples	C (%)	O (%)	N (%)	P (%)	Ca (%)	Total (%)
PMSs	57.31	42.69	-	-	-	100
aminated-PMSs	42.36	30.44	27.20	-	-	100
BCP-M-PMSs	54.14	43.51	-	0.99	1.36	100
BCP-IM-PMSs	44.52	38.96	11.84	1.90	2.78	100

**Table 2:** Surface elemental compositions of PMSs, aminated PMSs, BCP-M-PMSs, and BCP-IM-PMSs.

Samples	C1s (%)	O1s (%)	N1s (%)	P2p (%)	Ca2p (%)	Total (%)
PMSs	73.96	26.04	-	-	-	100
aminated-PMSs	56.48	34.80	8.72	-	-	100
BCP-M-PMSs	63.54	33.68	-	0.87	1.91	100
BCP-IM-PMSs	61.12	28.55	5.44	1.02	3.87	100
